# Multi-context blind source separation by error-gated Hebbian rule

**DOI:** 10.1038/s41598-019-43423-z

**Published:** 2019-05-09

**Authors:** Takuya Isomura, Taro Toyoizumi

**Affiliations:** 1 Laboratory for Neural Computation and Adaptation, RIKEN Center for Brain Science, Wako, Saitama 351-0198 Japan; 2 RIKEN CBS-OMRON Collaboration Center, Wako, Saitama 351-0198 Japan

**Keywords:** Machine learning, Learning algorithms

## Abstract

Animals need to adjust their inferences according to the context they are in. This is required for the multi-context blind source separation (BSS) task, where an agent needs to infer hidden sources from their context-dependent mixtures. The agent is expected to invert this mixing process for all contexts. Here, we show that a neural network that implements the *error-gated Hebbian rule* (EGHR) with sufficiently redundant sensory inputs can successfully learn this task. After training, the network can perform the multi-context BSS without further updating synapses, by retaining memories of all experienced contexts. This demonstrates an attractive use of the EGHR for dimensionality reduction by extracting low-dimensional sources across contexts. Finally, if there is a common feature shared across contexts, the EGHR can extract it and generalize the task to even inexperienced contexts. The results highlight the utility of the EGHR as a model for perceptual adaptation in animals.

## Introduction

Inference of the causes of a sensory input is one of the most essential abilities of animals^1–3^ — a famous example is the cocktail party effect, i.e., the ability of a partygoer to distinguish a particular speaker’s voice against a background of crowd noise^4,5^. This ability has been modelled by blind source separation (BSS) algorithms^6,7^, by considering that several hidden sources (speakers) independently generate signal trains (voices), while an agent receives mixtures of signals as sensory inputs. A neural network, possibly inside the brain, can invert this mixing process and separate these sensory inputs into hidden sources using a BSS algorithm. Independent component analysis (ICA), achieves BSS by minimizing the dependency between output units^8,9^. Numerous ICA algorithms have been proposed for both rate-coding^10–13^ and spiking neural networks^14^.

Previously, we developed a biologically plausible ICA algorithm, referred to as the *error-gated Hebbian rule (EGHR)*^15^. This learning rule can robustly estimate the hidden sources that generate sensory data without supervised signals. Importantly, it can reliably perform ICA in undercomplete conditions^16^, where the number of inputs is greater than that of outputs. A simple extension of the EGHR can separate sources while removing noise within a single-layer neural network^17^, by simultaneously performing principal component analysis (PCA)^18,19^ and ICA. The EGHR is expressed as a product of pre- and post-synaptic neuronal activities and a third modulatory factor, each of which can be computed locally (i.e., local learning rule^16^). In this sense, the EGHR is more biologically plausible than non-local engineering ICA algorithms^10–12^. Because of these desirable properties, the EGHR is considered as a candidate mechanism for neurobiological BSS^20–22^, as well as a next-generation neuromorphic implementation^23,24^ for energy efficient BSS.

The optimal inference and behavior often depend on context. Indeed, our perception and decisions reflect this context dependency, i.e., cognitive flexibility^25^. Studies in primates have suggested that a contextual-cue-dependent dynamic process in the prefrontal cortex controls this behavior^26–28^, and several computational studies have modeled it^29–32^. Likewise, context dependence of auditory perceptual inference has been modeled^33^. In addition to experimental evidence, recent progress in machine learning has also addressed this multi-context problem, in an attempt to create artificial general intelligence^34–36^. By implementing (task-specific) synaptic consolidation, a neural network can learn a new environment, while retaining past memories, by protecting synaptic strengths that are important to memorizing past environments. Those findings indicate the importance of multi-context processes for cognitive flexibility.

Unlike the above-mentioned tasks, BSS in several different contexts has some difficulty. Conventional ICA algorithms assume the same number of input and output neurons^10–12,37,38^ and cannot straightforwardly perform a multi-context BSS. After learning, the synaptic strength matrix of these algorithms converges to the inverse of the mixing matrix of the current context (or its permutation or sign-flip), which is generally different from that in the previous context. Hence, when the network subsequently encounters a previously learnt context, it needs to relearn the synaptic strengths from the very beginning. More involved engineering ICA algorithms, such as the non-holonomic ICA algorithm^39^ and the ICA mixture algorithm^40,41^, are expected to perform the multi-context BSS. However, a biological implementation of these non-local learning rules is unclear. Further, as we show below, they cannot learn to compress redundant inputs by extracting the underlying low-dimensional hidden sources.

Here we show that the EGHR can perform multi-context BSS when a neural network receives redundant sensory inputs. It can retain memories of previously experienced contexts and process the BSS right after contextual switching to a previously learnt context. This suggests that the EGHR can also be used as a powerful data compression method^42^, since it extracts low-dimensional hidden sources across contexts, despite the proportional increase in data dimensions to the number of contexts. Moreover, when a common feature is shared across contexts, the EGHR can extract it to perform BSS, while filtering out features that vary among contexts. Once the learning is achieved, the network can perform BSS even in an inexperienced context, indicating some generalization capability or transfer learning. We demonstrate that the EGHR with sufficiently redundant sensory inputs learns to distinguish birdsongs from their superpositions and retains this ability even after learning different sets of birdsongs. The rule finds a general representation that is capable of separating an unheard set of birdsongs. Finally, possible neurobiological implementations of the EGHR are discussed.

## Results

### Error-gated Hebbian rule (EGHR)

**Figure 1:**
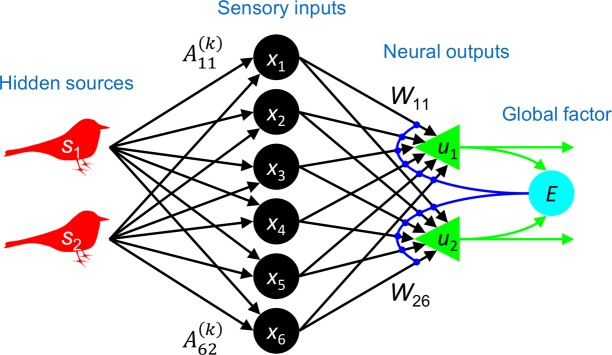
Model setup for multi-context BSS task. In this model, ${s}_{1},\ldots ,{s}_{{N}_{s}}$ are hidden sources (e.g., birdsongs); ${x}_{1},\ldots ,{x}_{{N}_{x}}$ are sensory inputs that an agent receives; ${u}_{1},\ldots ,{u}_{{N}_{u}}$ are neural outputs; ${A}_{11}^{(k)},\ldots ,{A}_{1{N}_{s}}^{(k)},{A}_{21}^{(k)},\ldots ,{A}_{{N}_{x}{N}_{s}}^{(k)}$ are elements of the *k*-th-context mixing matrix; ${W}_{11},\ldots ,{W}_{1{N}_{x}},{W}_{21},\ldots ,{W}_{{N}_{u}{N}_{x}}$ are synaptic strengths; and *E* is a scalar global factor that mediates synaptic plasticity. Synaptic strengths are adjusted to perform multi-context BSS by the EGHR.

In a BSS task, several hidden sources (*s*) independently generate signal traces, while our agent receives their mixtures as sensory inputs (*x*). In this study, we considered a multi-context BSS task, in which a set of contexts with different mixing weights was used. Sensory inputs were randomly generated from one of these contexts for a period of time, with *k* (=1, …, *C*) being an index of context. Our experimental setup consisted of an *N*_*s*_-dimensional vector of hidden sources $s\equiv {({s}_{1},\ldots ,{s}_{{N}_{s}})}^{T}$ whose elements *s*_*i*_ independently follow a non-Gaussian distribution *p*(*s*_*i*_), an *N*_*x*_-dimensional vector of sensory inputs $x\equiv {({x}_{1},\ldots ,{x}_{{N}_{x}})}^{T}$, and an *N*_*u*_-dimensional vector of neural outputs $u\equiv {({u}_{1},\ldots ,{u}_{{N}_{u}})}^{T}$ (Fig. 1). The sensory inputs in the *k*-th condition were generated by transforming the hidden sources, i.e., the so-called generative process:

1 $$\begin{array}{c}{\bf{S}}{\bf{e}}{\bf{n}}{\bf{s}}{\bf{o}}{\bf{r}}{\bf{y}}\,{\bf{i}}{\bf{n}}{\bf{p}}{\bf{u}}{\bf{t}}{\bf{s}}\,\,x={A}^{(k)}s.\end{array}$$

Here *A*^(*k*)^ is the *N*_*x*_ × *N*_*s*_ mixing matrix for the *k*-th context that defines the magnitude of inputs, when each source generates a signal. To ensure that each *A*^(*k*)^ represents a different context and that each context has an ICA solution, column vectors of a block matrix (*A*^(1)^, *A*^(2)^, …, *A*^(*C*)^) are supposed to be linearly independent of each other. We designed the task such that these contexts appear sequentially or randomly. The neural outputs were expressed as sums of inputs weighted by an *N*_*u*_ × *N*_*x*_ synaptic strength matrix *W*, and calculated by:

2 $$\begin{array}{c}{\bf{N}}{\bf{e}}{\bf{u}}{\bf{r}}{\bf{a}}{\bf{l}}\,{\bf{o}}{\bf{u}}{\bf{t}}{\bf{p}}{\bf{u}}{\bf{t}}{\bf{s}}\,\,u=Wx.\end{array}$$

It is well known that when a presynaptic neuron (*x*_*j*_) and a postsynaptic neuron (*u*_*i*_) fire together, Hebbian plasticity occurs, and the synaptic connection from *x*_*j*_ to *u*_*i*_, denoted by *W*_*ij*_, is strengthened^43,44^. Because this constitutes associative learning, correlations between ${x}_{1},\ldots ,{x}_{{N}_{x}}$ and *u*_*i*_ are usually enhanced; thereby, correlations among neural outputs also increase. This process is distinct from separation of signals (i.e., BSS) for which each neural output is expected to encode a specific source. To separate signals, we introduced a global scalar factor (i.e., a third factor) given by the sum of nonlinearly-transformed output units^15^:

3 $$\begin{array}{c}{\bf{G}}{\bf{l}}{\bf{o}}{\bf{b}}{\bf{a}}{\bf{l}}\,{\bf{f}}{\bf{a}}{\bf{c}}{\bf{t}}{\bf{o}}{\bf{r}}\,\,E(u)\equiv -\,{\rm{l}}{\rm{o}}{\rm{g}}\,{p}_{0}(u)=-\,\sum _{i=1}^{{N}_{u}}\,{\rm{l}}{\rm{o}}{\rm{g}}\,{p}_{0}({u}_{i}).\end{array}$$

Here *p*_0_(*u*) is the prior distribution that the agent expects the hidden sources to follow; e.g., when *p*_0_(*u*) is a Laplace distribution of mean zero and unit variance, then $E(u)=\sqrt{2}(|{u}_{1}|+\ldots +|{u}_{{N}_{u}}|)+{\rm{const}}$. We supposed that this global factor modulates Hebbian plasticity. Recent experimental studies have reported that synaptic plasticity can be modulated by various neuromodulators^45–49^, GABAergic inputs^50,51^, or glial factors^52^. Possible neurobiological implementations of the global factor are further discussed in the Discussion section. Overall, the synaptic strength matrix *W* is updated by the EGHR in the following way:

4 $$\begin{array}{c}{\bf{S}}{\bf{y}}{\bf{n}}{\bf{a}}{\bf{p}}{\bf{t}}{\bf{i}}{\bf{c}}\,{\bf{p}}{\bf{l}}{\bf{a}}{\bf{s}}{\bf{t}}{\bf{i}}{\bf{c}}{\bf{i}}{\bf{t}}{\bf{y}}\,({\bf{E}}{\bf{G}}{\bf{H}}{\bf{R}})\,\,\dot{W}\propto \mathop{\underbrace{\langle ({E}_{0}-E(u))}}\limits_{{\rm{g}}{\rm{l}}{\rm{o}}{\rm{b}}{\rm{a}}{\rm{l}}\,{\rm{f}}{\rm{a}}{\rm{c}}{\rm{t}}{\rm{o}}{\rm{r}}}\,\mathop{\underbrace{g(u)}}\limits_{{\rm{p}}{\rm{o}}{\rm{s}}{\rm{t}}}\mathop{\underbrace{{x}^{T}\rangle }}\limits_{{\rm{p}}{\rm{r}}{\rm{e}}},\end{array}$$

where $\dot{W}$ with respect to time, 〈·〉 is the expectation over the input distribution, and *g*(*u*) ≡ d*E*(*u*)/d*u* is a non-linear function usually associated with a nonlinear activation function. A constant *E*_0_ scales the neural outputs; the output scale becomes equivalent to the source scale when ${E}_{0}=\langle \,-\,{\rm{l}}{\rm{o}}{\rm{g}}\,{p}_{0}(s)\rangle +1$. In short, the EGHR constitutes a Hebbian learning rule when the global factor is smaller than the threshold (*E*(*u*) < *E*_0_); otherwise (*E*(*u*) > *E*_0_), it becomes an anti-Hebbian rule. This mechanism makes output neurons independent from each other. The detailed derivation and theoretical proofs of the EGHR have been described in our previous reports^15,17^. Briefly, the EGHR is derived as the gradient descent of the cost function *L* ≡ 〈(*E*(*u*) − *E*_0_)^2^〉/2. This is the cost for having dependency among outputs, designed for measuring the nonlinear correlation among elements of *u*. Hence, the minimization of *L* makes the elements of *u* independent of each other. The formal relationship between the EGHR and ICA algorithm based on the infomax principle is described in^17^.

### Memory capacity of the EGHR

First, we analytically show the memory capacity of a neural network established by the EGHR. As the number of contexts increases, larger dimensions of inputs are needed to retain information pertaining to past contexts in the neural network. For simplicity, we supposed that *N*_*u*_ = *N*_*s*_. Because the network represents a linear inverse model of the generative processes, the goal of the multi-context BSS is generally given by:

5 $$\begin{array}{c}W({A}^{(1)},\,\ldots ,\,{A}^{(C)})=({{\rm{\Omega }}}^{(1)},\,\ldots ,\,{{\rm{\Omega }}}^{(C)}),\end{array}$$

where Ω^(*k*)^ is an *N*_*u*_ × *N*_*s*_ matrix equivalent to the identity matrix, up to permutations and sign-flips. This is because the success of BSS is defined by one-by-one mapping from sources to outputs. Thus, the multi-context BSS is successful if and only if a set of mixing matrices (*A*^(1)^, …, *A*^(*C*)^) expresses a full-column-rank matrix (see Methods for the derivation). Hence, we found that the following conditions are necessary to achieve the multi-context BSS for a generic (*A*^(1)^, …, *A*^(*C*)^): (1) the input dimension needs to be equal to or larger than the number of contexts times the number of sources, *N*_*x*_ ≥ *CN*_*s*_; and (2) the output dimension needs to be equal to or larger than the source dimension, *N*_*u*_ ≥ *N*_*s*_. Note that the neural network learns the information representation that compresses the sensory inputs, because we considered the input dimensions that are much greater than the output dimensions.

**Figure 2:**
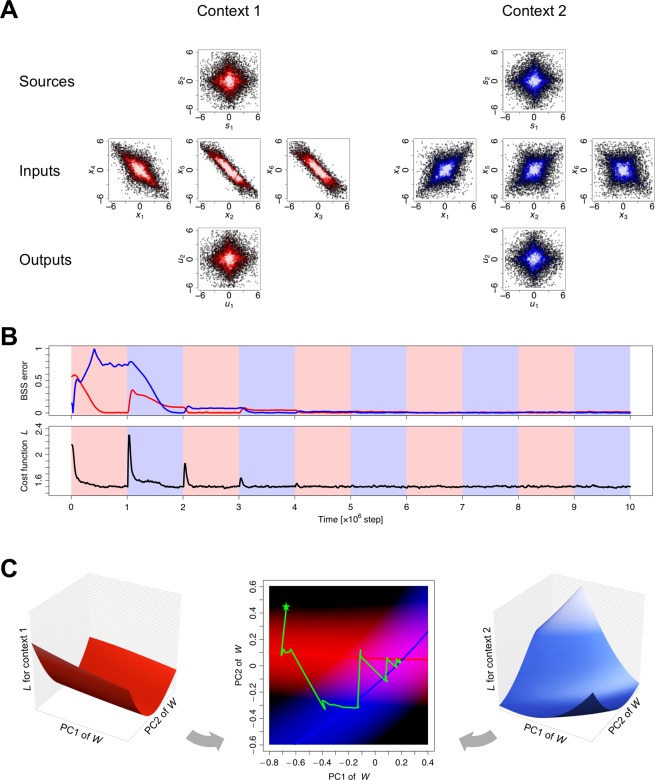
Results of multi-context BSS. (**A**) Distributions of sources, inputs, and outputs for context 1 and 2. (**B**) Trajectories of BSS error in the two contexts (top, red: context 1, blue: context 2) and cost function (bottom). (**C**) Visualization of null spaces. The panel illustrates the shapes of the cost function under each context (left and right) and the trajectory of synaptic strengths (*W*) projected in a subspace spanned by the first (PC1) and second (PC2) principal components (center). The trajectory is determined by the gradient of either cost function, depending on the context. On the PC1-PC2 plane, null spaces are illustrated as nullclines; red and blue curves are nullclines for contexts 1 and 2, respectively. Low cost areas (i.e., valleys of the cost functions) are highlighted by red or blue shading. The synaptic strength matrix starts from a random initial state (star mark), shifts to the nullcline of context 1 or 2, and eventually converges to the cross point of the two nullclines, where the synaptic strengths perform the BSS for both contexts. Each source was randomly generated by the unit Laplace distribution. A learning rate of *η* = 4 × 10^−6^ was used. The MATLAB source code for this simulation is appended as Supplementary Source Codes.

The memory capacity of the EGHR was empirically confirmed by numerical simulations (Fig. 2). Here, we supposed that two contexts generated inputs alternately. In each context, six-dimensional inputs were generated from two-dimensional sources with different mixing weights, as denoted by *A*^(1)^ and *A*^(2)^ (see top and middle rows in Fig. 2A). A neural network consisting of six input and two output neurons received the inputs and changed its synaptic strengths through the EGHR (i.e., training). After training, each neural output came to selectively respond to (i.e., encode) one of the two sources (bottom row in Fig. 2A). Thus, the network achieved separation of the sensory inputs into their sources without being taught the mixing weights (i.e., BSS).

Crucially, the neural network was able to retain the information learnt for all past contexts if provided with sufficiently redundant sensory inputs. This property is illustrated by the trajectories of the BSS error and EGHR cost function in Fig. 2B. We defined the BSS error for context *k* as the ratio of first to second maximum absolute values averaged for every row and column of matrix *K*^(*k*)^ ≡ *WA*^(*k*)^ (see Methods for the mathematical definition of the BSS error). Here *K*^(*k*)^ expresses the mapping from sources to outputs, which is equivalent to the covariance matrix between hidden sources and neural outputs *K*^(*k*)^ = Cov(*u*, *s*). This definition of the BSS error was made to ensure that the value was zero if and only if one source mapped onto one output, and *vice versa*; otherwise the value was positive and less than one. Moreover, the cost function of the EGHR was defined as the expectation of the square of the global factor: *L* = 〈(*E*_0_ − *E*(*u*))^2^〉/2. Context 1 (red in Fig. 2A) was provided in the first session. Since synaptic strengths started from a random initial state, the BSS error at the beginning of the first session was large; then, the network learned an optimal set of synaptic strengths, and the error became zero, which was achieved by minimizing the cost function through gradient descent updates. When context 2 (blue in Fig. 2A) was provided for the first time in the second session, the EGHR cost function transiently increased, as it needed to learn the new mixing matrix. An important point was revealed at the first step of the third session, in which context 1 was provided again. The BSS error was significantly smaller than that in the first session and close to zero from the beginning of this session, indicating that the network retained synaptic strengths that were optimized for context 1 even after learning context 2. After several iterations, the BSS error for both contexts converged to zero. The success of learning was also confirmed by the trajectory of the EGHR cost function that also converged to the minimum value (Fig. 2B bottom).

These results show that an undercomplete EGHR increased the speed of re-adaptation to previously experienced contexts, suggesting that memory of past experiences was preserved within the network. Moreover, the network learned the optimal set of synaptic strengths that entertained both contexts after several iterations. A key feature for this ability is the “null space” in the synaptic strength matrix. While only four (2 × 2) dimensions were required to express a mapping from two-dimensional sources to two-dimensional outputs in one context, the synaptic strength matrix still comprised eight (2 × 6 − 2 × 2)-dimensional degrees of freedom. This freedom spanned a null space in which synaptic strengths were equally optimized with zero BSS error. Similarly, when two different contexts were considered, four-dimensional degrees of freedom remained, as an overlap between the two eight-dimensional null spaces. To visualize such a null space, we projected synaptic strengths onto a subspace spanned by the first (PC1) and second (PC2) principal components of the trajectory of synaptic strengths (Fig. 2C). On this PC1-PC2 plane, a null space was illustrated as a nullcline. Since the dynamics of synaptic strengths were determined to go down the slope of a cost function for either context 1 or 2, synaptic strengths were started from a random initial state and reached the nullcline of either context 1 or 2, in turn. Crucially, this trajectory converged to the cross point of the two nullclines, where the synaptic strengths entertained both contexts. Because of this, the BSS error reached zero after iterative training; i.e., the network solved ICA for both contexts.

**Figure 3:**
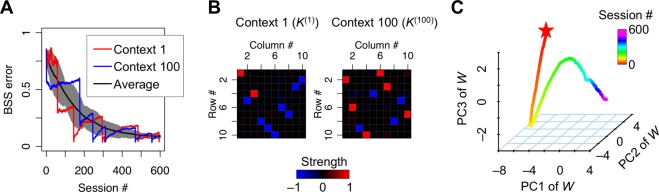
BSS with large number of contexts. One of 100 different contexts was randomly selected for each session. Each session contained *T* = 10^5^ time steps, and the training continued for 600 sessions. In each session, the 2000-dimensional sensory inputs (*x*) were generated from ten-dimensional hidden sources (*s*), which independently followed the unit Laplace distribution, through a context-dependent random mixing matrix *A*^(*k*)^. The neural network consisting of ten-dimensional neural outputs (*u*) was trained with a learning rate of *η* = 10^−5^. (**A**) Trajectories of BSS error for context 1 and 100 and the average BSS error over contexts 1 to 100. The shaded area shows the standard deviation. (**B**) Mappings from ten sources to ten outputs in contexts 1 and 100 after training. Elements of matrix *K*^(*k*)^ = *W A*^(*k*)^ with *k* = 1 and 100 are illustrated by the heat map. Only one element in each row and column takes ±1, indicating the one-to-one mapping from sources to outputs, i.e., the success of multi-context BSS. (**C**) The dynamics of synaptic strength matrix *W* projected in the three-dimensional space spanned by the first to third principal components (PC1 to PC3). The matrix starts from a random initial point (star mark) and converges to the null space, in which synaptic strengths are optimized for all trained contexts. The C code for this simulation is appended as Supplementary Source Codes.

Furthermore, we examined the multi-context BSS by the EGHR using a large number of contexts (Fig. 3). Our agent received redundant (2000-dimensional) sensory inputs, comprising 100 sets (contexts) of mixtures of ten hidden sources (1000 sources in total), that were generated as products of the context-dependent mixing matrix and sources. Ten outputs neurons learned to infer each source from their mixtures by updating synaptic strengths through the EGHR. After training, we found that they successfully represented the ten sources for every context, without further updating synaptic strengths, as illustrated by the reduction of the BSS error for all 100 contexts (Fig. 3A) and the convergence of the covariance between sources and outputs to a diagonal matrix (up to permutations and sign-flips) (Fig. 3B). This was because synaptic strengths had sufficient capacity and were formed to express the inverse of the concatenated mixing matrices from all contexts, which was further confirmed by the convergence of the synaptic strength matrix in the null space (Fig. 3C).

### BSS in constantly time-varying environments

In the previous section, we described a general condition for the neural network to achieve the multi-context BSS. In special cases, where the mixing matrices in each context have common features, the neural network can perform the multi-context BSS beyond the maximum number of contexts described above. Here, we show that when contexts are generated from a low-dimensional subspace of mixing matrices and, therefore, are dependent on each other, the EGHR can find the common features and use them to perform the multi-context BSS.

**Figure 4:**
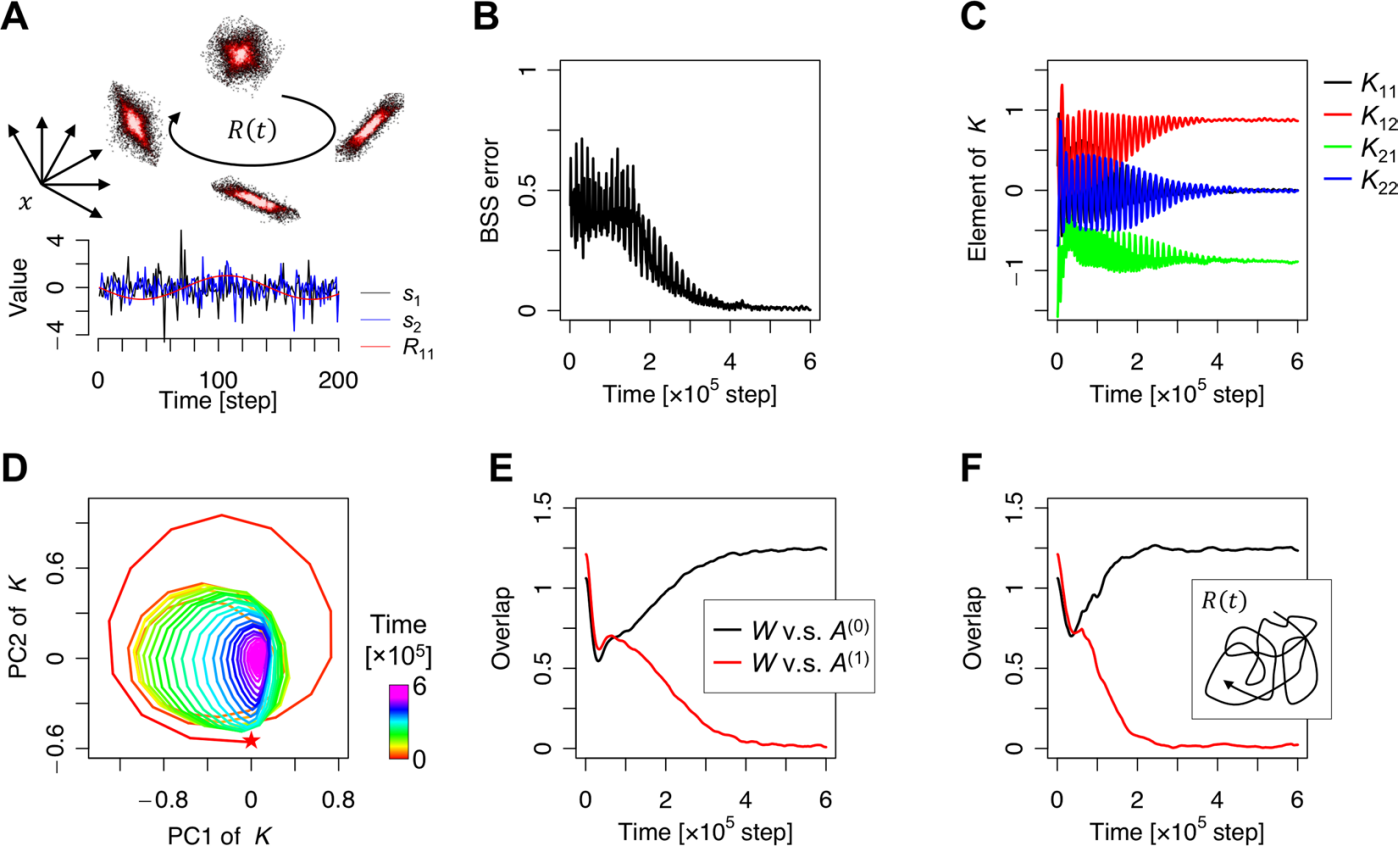
BSS with time-varying mixing matrix. (**A**) Top: Schematic image of sensory inputs generated from two sources through time-varying mixing matrix *A*(*t*). The mixing matrix is controlled by the low-dimensional rotation matrix *R*(*t*). Bottom: Trajectories of hidden sources and an element of *R*(*t*), showing the difference in their time courses. (**B**) Trajectory of BSS error. (**C**) Trajectories of mapping weights from sources to outputs, i.e., matrix *K* = *W A*(*t*). (**D**) Dynamics of matrix *K* projected on the first two-dimensional PCA subspace of *K*’s trajectory over training. The matrix starts from a random initial point (star mark) and follows a spiral trajectory as it converges to a subspace in which synaptic matrix *W* is perpendicular to the time-varying component *A*^(1)^. (**E**) Overlap of synaptic matrix *W* with time-invariant component *A*^(0)^ and time-variant component *A*^(1)^. The overlap between two matrices was defined by the Frobenius norm of their product, i.e., ${|W{A}^{(k)}|}_{F}\equiv \sqrt{\sum _{ij}{(W{A}^{(k)})}_{ij}^{2}}$. (**F**) Overlap of synaptic matrix *W* with *A*^(0)^ and *A*^(1)^ when elements of *R*(*t*) were modeled as OU processes. Each source was randomly generated by the unit Laplace distribution. The learning rate of *η* = 10^−5^ was used. The C code for this simulation is appended as Supplementary Source Codes.

As a corollary of the property of the EGHR when provided with redundant inputs, the EGHR can perform the BSS even when the mixing matrix changes constantly as a function of time (Fig. 4A). Without loss of generality, a time-dependent mixing matrix is expressed by the sum of time-invariant and time-variant components, as follows:

6 $$\begin{array}{c}A(t)\equiv \mathop{\underbrace{{A}^{(0)}}}\limits_{{\rm{time}} \mbox{-} {\rm{invariant}}}+\mathop{\underbrace{{A}^{(1)}R(t)}}\limits_{{\rm{time}} \mbox{-} {\rm{variant}}},\end{array}$$

where *A*^(0)^ is a full-column-rank constant matrix with the same size as *A*(*t*), *A*^(1)^ is a full-column-rank constant vertically-long rectangular (or square) matrix, and *R*(*t*) is a matrix composed of either smoothly or discontinuously changing functions in time. Each component of *R*(*t*) is supposed to on average slowly change, i.e., their time-derivatives are typically much smaller in magnitude than those of *s*(*t*). This condition is required to distinguish whether changes in inputs are caused by changes in the mixing matrix *A*(*t*) or the hidden sources *s*(*t*). Formally, *A*(*t*) expresses infinite contexts along the trajectory of *R*(*t*). This is a more complicated setup than the standard BSS in the sense that both sources and the mixing matrix change in time. Nonetheless, the EGHR can achieve BSS for all contexts if a solution of the synaptic strength matrix that satisfies *W*(*A*^(0)^, *A*^(1)^) = (Ω, *O*) exists. Here, Ω represents the identity matrix up to permutations and sign-flips and *O* represents a matrix with zero elements. Such a solution generally exists if and only if (*A*^(0)^, *A*^(1)^) is a full-column-rank matrix (see Methods for the derivation). The above condition means that the network performs BSS based on the time-invariant features *A*^(0)^ of the mixing matrix, while neglecting the time-varying features *A*^(1)^*R*(*t*). This can be viewed as a way to compress high-dimensional data. This is distinct from the standard dimensionality reduction approach by PCA, which would preferentially extract the time-variant features due to their extra variances. Moreover, the ability to perform dimensionality reduction is an important advantage of the EGHR over conventional ICA algorithms, such as the infomax-based ICA^10,11^, natural gradient^12^ and nonholonomic^39^ algorithms, and the ICA mixture model^40^, because these learning algorithms do not learn effective dimensionality reduction in the multi-context BSS setup due to their construction (see Methods for mathematical explanations).

In the simulation, we supposed *R*(*t*) to be a two-dimensional rotation matrix, $$R(t)=(\cos \,\omega t,-\,\sin \,\omega t;$$$$\sin \,\omega t,\,\cos \,\omega t)$$, with an angular frequency of $$\omega =\sqrt{2}\pi /100$$. The simulation showed a reduction in the BSS error (Fig. 4B). At the same time, *K* = *WA* converged to the identity matrix up to permutations and sign-flips, *K* → (0,1; −1,0) in this case, although *A* continuously changed in time (Fig. 4C,D). As illustrated in Fig. 4E, the synaptic matrix *W* became perpendicular to the time-varying features *A*^(1)^ (i.e., *WA*^(1)^ = *O*), by a monotonic reduction of the overlap between *W* and *A*^(1)^ (defined by the Frobenius norm of their product). After training, the overlap converged to zero. Hence, synaptic strengths were optimized regardless of *R*(*t*) at this solution, which enabled the network to perform BSS with a virtually infinite number of contexts. In addition, the neural network that implements the EGHR could learn *W* perpendicular to *A*^(1)^ in another simulation setup, where *R*(*t*) was a 2 × 2 matrix and its elements were modeled as Ornstein-Uhlenbeck (OU) processes with time constant *τ* = 10^−3^ (Fig. 4F). These results indicate that the EGHR can perform the multi-context BSS with a wide range of time-varying mixing matrices. Indeed, a mathematical analysis shows that multi-context BSS is possible for a general time-varying matrix *R*(*t*) as long as it changes slowly enough (see Methods).

**Figure 5:**
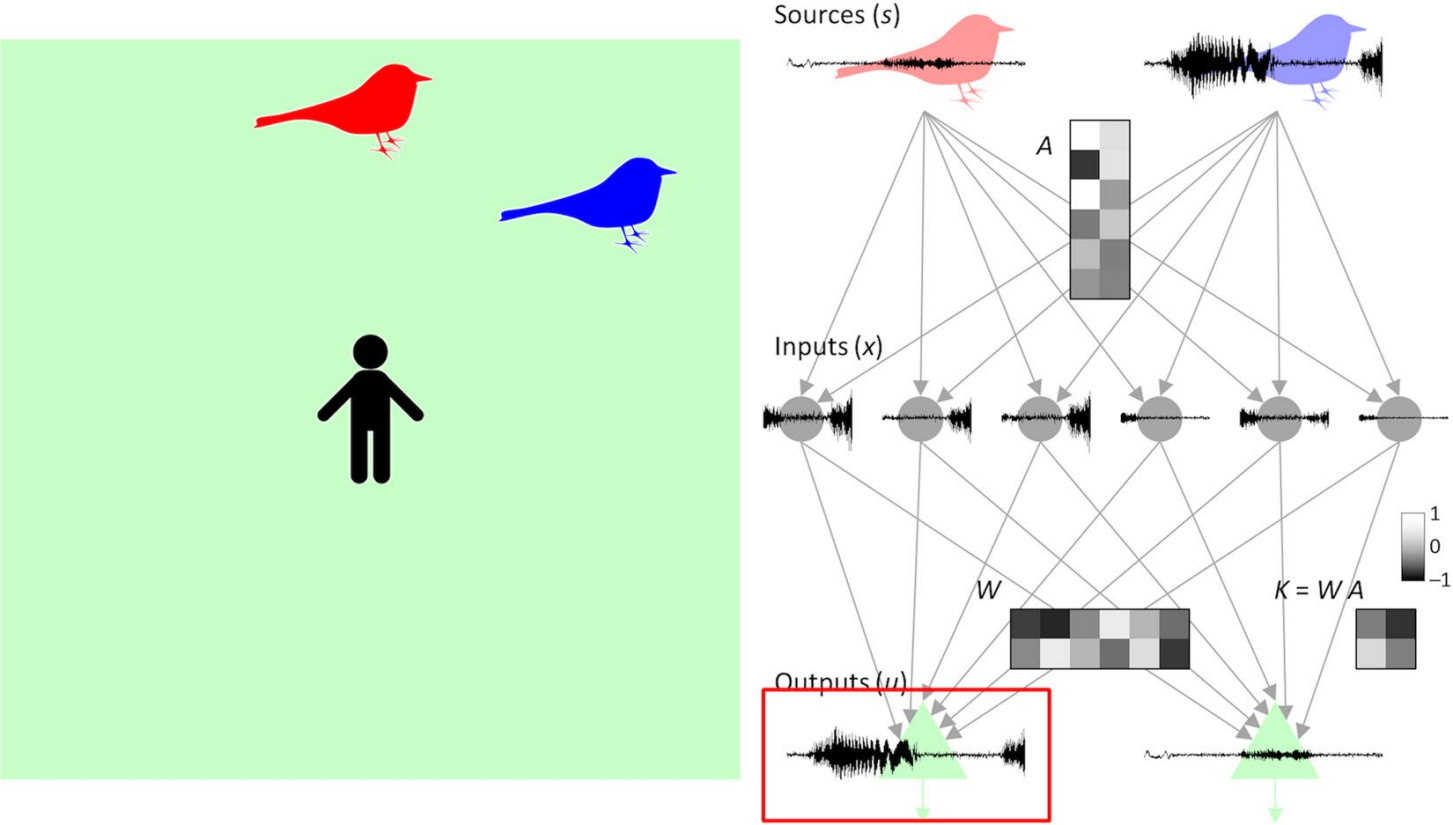
BSS of birdsongs when two birds move around the agent. A snapshot of the simulation overview movie after training is shown. Songs (or sources) generated by two birds *s*_1_, *s*_2_ (right top) are mixed with a time-varying mixing matrix *A*, resulting in six-dimensional sensory inputs *x*_1_, …, *x*_6_ (right middle). The mixed signals correspond to the recording through six microphones with different preferences. The neural network converts the six inputs into two neural outputs, *u*_1_ and *u*_2_ (right bottom), using synaptic strength matrix *W*. The synaptic updates by the EGHR enable the outputs to encode each birdsong. Matrix *K* = *W A* represents the mapping from sources to outputs. See Methods for the detailed simulation setup.

Next, we demonstrated the utility of the EGHR, when supplied with redundant inputs, by using natural birdsongs and a time-variant mixing matrix that expressed a natural contextual change. Figure 5 illustrates the BSS task of two birdsongs when birds moved around the agent; thereby, the mixing matrix changed in time according to the positions of the birds (see, the entire movie at http://toyoizumilab.brain.riken.jp/dataset/Isomura2019/Isomura_Toyoizumi_SciRep2019_SupplementaryMovieS1.mp4). To obtain time-independent features, we assumed that the two birds moved around in non-overlapping areas. For simplicity, we also assumed that the two birds moved around at different heights. The agent received mixtures of the two birdsongs through six microphones with different direction preferences. In the current context, the z-axis of the birds was time-invariant and the x- and y-axes of the birds were time-variant, although the observer was not informed about this. By tuning synaptic strengths by the EGHR, neural outputs were established to infer each birdsong, while the mixing matrix changed continuously. Crucially, after training, the mapping from the sources to the outputs (*K* = *W A*) became constant with time, although matrix *A* was time-dependent. More precisely, the EGHR found a representation where *W* satisfied *W*(*A*^(0)^, *A*^(1)^) = (Ω, *O*). Hence, neural outputs could separate the two birdsongs, although the amplitudes of the songs recorded by the microphones continuously changed depending on the positions of birds.

### Generalization for inexperienced environments

Finally, we examined the generalization capability of the multi-context BSS by the EGHR using natural birdsongs. For the sake of simplicity, we reduced Eq. (6) by considering *R*(*t*) that changes discontinuously at the beginning of each session but otherwise is constant. Specifically, we considered the mixing matrix

7 $$\begin{array}{c}A(v)=\mathop{\underbrace{{A}^{(0)}}}\limits_{{\rm{context}} \mbox{-} {\rm{independent}}}+\mathop{\underbrace{{A}^{(1)}{v}_{1}+\cdots +{A}^{(n)}{v}_{n}}}\limits_{{\rm{context}} \mbox{-} {\rm{dependent}}},\end{array}$$

written using context-independent matrix *A*^(0)^, context-dependent matrices {*A*^(1)^, …, *A*^(*n*)^}, and context vector *v* ≡ (*v*_1_, …, *v*_*n*_) that discontinuously changes at the beginning of a new session. The first term in the right-hand side of Eq. (7) corresponds to the context-independent (i.e., constant) component, which should be a full-column-rank matrix to provide an ICA solution. Similarly to the case with the continuously time-varying mixing matrix, the EGHR can establish synaptic matrix *W* that expresses the pseudo inverse of *A*^(0)^ up to permutations and sign-flips, while keeping *W* perpendicular to *A*^(1)^, …, *A*^(*n*)^, i.e., *W*(*A*^(0)^, *A*^(1)^, …, *A*^(*n*)^) = (Ω, *O*, …, *O*). Notably, the EGHR can establish such *W* by using only a handful samples of *v* out of combinatorially many possibilities. This is because the mappings from sources to inputs are restricted to be a linear transformation, and thereby, observations with the polynomial (probably quadratic) order number of contexts can identify the mapping for all contexts. This property is particularly useful when *v* is high dimensional.

**Figure 6:**
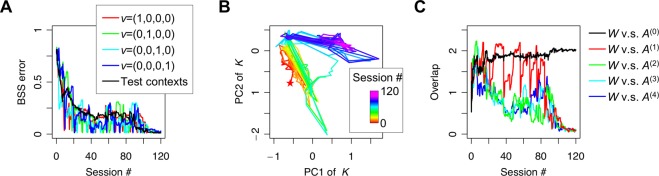
Generalization of multi-context BSS. (**A**) Trajectories of the BSS error with four trained contexts and the average BSS error over 20 inexperienced test contexts, created using randomly sampled *v*. (**B**) Dynamics of matrix *K* projected on the first two-dimensional PCA subspace. The matrix starts from a random initial point (star mark) and converges to a fixed point, at which the synaptic matrix entertains every trained context. (**C**) Overlap of synaptic matrix *W* with context-independent component *A*^(0)^ and context-dependent components *A*^(1)^, …, *A*^(4)^. Overlap between two matrices is defined by |*WA*^(*k*)^|_*F*_, as described in Fig. 4. See Methods for the detailed simulation setup.

In this demonstration, ten sets (contexts) of mixtures of ten birdsongs were introduced to our agent, with redundant sensory inputs composed of 100 mixed sound waves (Fig. 6). Those contexts were defined by random mixing matrices *A*^(0)^, *A*^(1)^, …, *A*^(4)^. We trained the network using only 10 contexts: *v* = (1,0,0,0), (½,½,0,0), (0,1,0,0), (0,½,½,0), (0,0,1,0), (0,0,½,½), (0,0,0,1), (½,0,0,½), (½,0,½,0), (0,½,0,½). At the beginning of each session, *v* was randomly selected from the above ten vectors, which provided a discrete random transition among 10 contexts. Ten output neurons learned to infer each birdsong from their mixtures, by updating synaptic strengths through the EGHR. After training, they successfully represented the ten birdsongs without further updating synaptic strengths. Crucially, the network could perform BSS even in an inexperienced context (for example, in *v* = (¼,¼,¼,¼)). This speaks to the generalization of the multi-context BSS for unseen test contexts.

We quantitatively showed that, as learning progresses, the BSS error for test contexts (defined using 20 randomly sampled *v* that were inexperienced in the training), as well as for trained contexts, decreased (Fig. 6A). The trajectory of the first two principal components (PC1 and PC2) of *K* exhibits their convergence to a fixed point at later sessions (Fig. 6B). Here PC1 and PC2 together captured 63.4% of the total variance. Regardless of the given context, matrix *K* converged to a constant matrix that was the same as the identity matrix up to permutations and sign-flips. The convergence of *W* to this fixed point was validated by plotting the trajectories of the overlaps between *W* and *A* components (Fig. 6C). While the overlap between *W* and *A*^(0)^ increased as learning progressed, the overlap with context-dependent components (*A*^(1)^, …, *A*^(4)^) decreased and converged to zero, showing that *W* became perpendicular to *A*^(1)^, …, *A*^(4)^ by the EGHR. We conducted a series of simulations with different initial conditions and confirmed the reliability of convergence, although the convergence speed depended on the initial relative position of *W* compared to *A*^(0)^, *A*^(1)^, …, *A*^(4)^. Hence, this learnt network could perform BSS with *A*(*v*) determined by arbitrary *v* in the four-dimensional space, without further synaptic updating or transient error, while the network was trained only with 10 contexts. Those results highlight the significant generalization capability of the neural network established by the EGHR and the robustness against inexperienced environments for performing BSS.

## Discussion

While a real environment comprises several different contexts, humans and animals retain the experience of past contexts to perform well when they find themselves in the same context in the future. This ability is known as conservation of learning or cognitive flexibility^25^. Although analogous learning is likely to happen during BSS, the conventional biological BSS algorithms^37,38^ must forget the memory of past contexts to learn a new one. Thereby, when the agent subsequently encounters a previously experienced context, it needs to relearn it from the very beginning. We overcame this limitation by using the described algorithm, the EGHR. The crucial property of the EGHR is that when the number of inputs is larger than the number of sources, the synaptic matrix contains a null space in which synaptic strengths are equally optimized for performing BSS. Hence, with sufficiently redundant inputs, the EGHR can make the synaptic matrix optimal for every experienced context. This is an ability that the conventional biologically plausible BSS algorithms do not have, due to the constraint that the number of inputs and outputs must be equal^15^; however, we argue that this ability is crucial for animals to perceive and adapt to dynamically changing multi-context environments. It is also crucial for animals to generalize past learning to inexperienced contexts. We also found that, if there is a common feature shared across the training contexts, the EGHR can extract it and generalize the BSS result to inexperienced test contexts. This speaks to a generalization capability and transfer learning, implying the prevention of overfitting to a specific context; alternatively, one might see this as an extraction of a general concept across contexts. Therefore, we argue that the EGHR is a good candidate model for describing the neural mechanism of conservation of learning or cognitive flexibility for BSS.

Moreover, the process of extracting hidden sources in a multi-context BSS setup can be seen as a novel concept of dimensionality reduction^42^. If the dimensions of input are greater than the product of the number of sources and the number of contexts, the EGHR can extract the low-dimensional sources (up to context-dependent permutations and sign-flips), while filtering out a large number of context-dependent signals induced by changes in the mixing matrix. ICA algorithms for multi-context BSS^39–41^ and undercomplete ICA for compressing data dimensionality^15,17,53^ have been separately developed. Nevertheless, conventional ICA algorithms for multi-context BSS cannot learn efficient dimensionality reduction, and thus, to our knowledge, our study is the first to attempt dimensionality reduction in the multi-context BSS setup. This method is particularly powerful when a common feature is shared across the contexts, because the EGHR can make each neuron encode an identical source across all contexts. Our results are different from those obtained using standard dimensionality reduction approaches by PCA^18,19^, because PCA is used for extracting subspaces of high-variance principal components and hence would preferentially extract the context-dependent varying features, given that each source has the same variance. Therefore, our study proposes an attractive use of the EGHR for dimensionality reduction.

It is worth noting that the application of standard ICA algorithms to high-pass filtered inputs cannot solve the multi-context BSS problem. This is because context-dependent changes in the mixing matrix not only change the means of the inputs, which can be removed by high-pass filtering, but also change the gain of how fluctuations of each source are propagated to input fluctuations. Hence, the difference in contexts cannot be expressed as a linear ICA problem after high-pass input filtering. Therefore, selective extraction of context-invariant features is an advantage of the EGHR. Moreover, if provided with redundant input, the EGHR can solve multi-context BSS even if the context changes continuously in time, as we demonstrated in Figs. 4, 5.

We demonstrated that a neural network learns to distinguish individual birdsongs from their superposition. Young songbirds learn songs by mimicking adult birds’ songs^54–57^. A study reported that neurons in songbirds’ higher auditory cortex exhibit a teacher specific activity^58^. One can imagine those neurons correspond to the expectation of hidden sources (*u*), as considered in this study. Importantly, the natural environment that young songbirds encounter is dynamic, as we considered in Fig. 5. Therefore, the conventional BSS setup, which assumes a static environment or context, is not suitable for explaining this problem. It is interesting to consider that young songbirds might employ some computational mechanism similar to the EGHR to distinguish a teacher’s song from other songs in a dynamically changing environment.

Biological neural networks implement an EGHR-like learning rule. The main ingredients of the EGHR are Hebbian plasticity and the third scalar factor that modulates it. Hebbian plasticity occurs in the brain depending on the activity level^44,59^, spike timings^60–63^, or burst timings^64^ of pre- and post-synaptic neurons. In contrast, the third scalar factor can modify the learning rate and even invert Hebbian to anti-Hebbian plasticity^50^, similarly to what we propose for the EGHR. In general, such a modulation forms the basis of a three-factor learning rule, a concept that has recently received attention (see^20,65,66^ for reviews), and is supported by experiments on various neuromodulators and neurotransmitters, such as dopamine^45–47^, noradrenaline^48,49^, muscarine^67^, and GABA^50,51^, as well as glial factors^52^. (These factors may encode reward^68–72^, likelihood^73^, novelty/surprise^74^, or error from a prior belief^15,17^ to achieve various types of learning, implying the existence of a unified three-factor learning framework.) Importantly, the EGHR only requires such a signal that conveys global information to neurons to achieve learning. Furthermore, a study using *in vitro* neural networks suggested that neurons perform simple BSS using a plasticity rule that is different from the most basic form of Hebbian plasticity, by which synaptic strengths are updated purely as a product of pre- and postsynaptic activity^75,76^. A candidate implementation of the EGHR can be made for cortical pyramidal cells and inhibitory neurons; the former constituting the EGHR output neurons and encoding the expectations of hidden sources, and the latter constituting the third scalar factor and calculating the nonlinear sum of activity in surrounding pyramidal cells. This view is consistent with the circuit structure reported for the visual cortex^77,78^. These empirical evidences support the biological plausibility of the EGHR as a candidate model of neuronal BSS.

A local computation of the EGHR is highly desirable for neuromorphic engineering^23,24,79,80^. The EGHR updates synapses by a simple product of pre- and postsynaptic neurons’ activity and a global scalar factor. Because of this, less information transfer between neurons is required, compared to conventional ICA methods that require non-local information^10–12^, all-to-all plastic lateral inhibition between output neurons^37,38^, or an additional processing step for decorrelation^13^. The simplicity of the EGHR is a great advantage when implemented in a neuromorphic chip because it can reduce the space for wiring and the energy consumption. Furthermore, unlike the conventional ICA algorithms that assume an equal number of input and output neurons, a neuromorphic chip that employs the EGHR with redundant inputs would perform BSS in multiple contexts, as allowed by the network memory capacity, without requiring readaptation. The generalization capability of the EGHR, as demonstrated in Fig. 6, is an additional benefit, as the EGHR captures the common features shared across training contexts to perform BSS in inexperienced test contexts.

Notably, although we considered a linear BSS problem in this study, multi-context BSS can be extended to non-linear BSS, in which the inputs are generated through a non-linear mixture of sources^81,82^. To solve this problem, a promising approach would be to use a linear neural network. A recent study showed that when the ratio of input-to-source dimensions and source number are large, a linear neural network can find an optimal linear encoder that separates the true sources through PCA and ICA, thus asymptotically achieving zero BSS error^83^. Because both the asymptotic linearization and multi-context BSS by the EGHR are based on high-dimensional sensory inputs, combining these two might be a useful approach to solve the multi-context and non-linear BSS problem.

In summary, we demonstrated that the EGHR can retain memories of past contexts and, once the learning is achieved for every context, it can perform multi-context BSS without further updating synapses. Moreover, the EGHR can find common features shared across contexts, if present, and uses them to generalize the learning result to inexperienced contexts. Therefore, the EGHR will be useful for understanding the neural mechanisms of flexible inference and sensory representation under dynamically changing environments, and for creating brain-inspired artificial general intelligence.

## Methods

### Model and learning rule

The neural network model and used learning rule (the EGHR) are described in the Results section.

### Definition of BSS error

We calculated the maximum and second maximum rows as $i^{\prime} ={{\rm{argmax}}}_{i}|{K}_{ij}^{(k)}|$ and $i^{\prime\prime} ={{\rm{argmax}}}_{i\ne i^{\prime} }|{K}_{ij}^{(k)}|$ and defined the BSS error of column *j* by the ratio of the values in the two rows: ${\varepsilon }_{j}^{c}=|{K}_{{i}^{^{\prime\prime} }j}^{(k)}|/|{K}_{i^{\prime} j}^{(k)}|$. Similarly, the BSS error of row *i*: ${\varepsilon }_{i}^{r}=|{K}_{i{j}^{^{\prime\prime} }}^{(k)}|/|{K}_{ij^{\prime} }^{(k)}|$ was obtained from the ratio of the maximum and second maximum columns, where $j^{\prime} ={{\rm{argmax}}}_{j}|{K}_{ij}^{(k)}|$ and $j^{\prime\prime} ={{\rm{argmax}}}_{j\ne j^{\prime} }|{K}_{ij}^{(k)}|$. The BSS error (for the whole *K*) was defined as the average of them: $BSS\,error\equiv \,({\varepsilon }_{1}^{c}+\ldots +{\varepsilon }_{{N}_{s}}^{c})/2{N}_{s}+({\varepsilon }_{1}^{r}+\ldots +{\varepsilon }_{{N}_{u}}^{r})/2{N}_{u}$.

### Analysis of BSS solution: existence and linear stability

Supposing that *N*_*u*_ = *N*_*s*_, we defined the transform matrix *K*^(*k*)^ by

8 $$\begin{array}{c}{K}^{(k)}\equiv W{A}^{(k)}.\end{array}$$

For *N*_*x*_ ≥ *N*_*s*_, the ICA for context *k* is achieved when *K*^(*k*)^ is the identical matrix up to permutations and sign-flips. Hence, when amd only when column vectors of a block matrix (*A*^(1)^, …, *A*^(*C*)^) are linearly independent of each other, i.e., if and only if (*A*^(1)^, …, *A*^(*C*)^) is a full-column-rank matrix, an ICA solution that separates all sources for context 1, …, *C* exists. Namely, *W* achieves the multi-context BSS when it satisfies

9 $$\begin{array}{c}({K}^{(1)},\ldots ,{K}^{(C)})=W({A}^{(1)},\ldots ,{A}^{(C)})=({{\rm{\Omega }}}^{(1)},\ldots ,{{\rm{\Omega }}}^{(C)}),\end{array}$$

where Ω^(*k*)^ is an *N*_*u*_ × *N*_*s*_ matrix equivalent to the identity matrix up to permutations and sign-flips. Regarding the *i*-th row of matrix *K*^(*k*)^, as denoted by a row vector $({K}_{i1}^{(k)},\ldots ,{K}_{i{N}_{s}}^{(k)})$, the achievement of ICA is justified when one element is one and the others are zero. Thus, there are many candidate sets of $({W}_{i1},\ldots ,{W}_{i{N}_{x}})$ that can achieve ICA, because *N*_*x*_ is larger than *N*_*s*_. Our numerical analyses showed that among these potential solutions, the one that is the nearest to the solution for the previous context is likely to be chosen. This can be understood as follows: when the network finds an ICA solution for all contexts, the error (i.e., cost function of the EGHR), including transient periods between two contexts, is minimized; hence, according to the gradient descent, synaptic strengths converge to such a solution as training progresses. Owing to this mechanism, the initial errors converge to zero when previously experienced environments are provided as stimuli.

We showed that *W* that satisfies *K* = *WA* = Ω gives a fixed point for the EGHR cost function, $\dot{W}=-\,\partial L/\partial W=({E}_{0}-E(u))g(u){x}^{T}=O$, and thus gives an ICA solution, where *A* is a vertically long or square full-rank mixing matrix^15,17^. Regarding BSS with a time-varying mixing matrix, from *A* = *A*^(0)^ + *A*^(1)^*R*, the time differential of *K* yields $\dot{K}=\dot{W}({A}^{(0)}+{A}^{(1)}R)+W{A}^{(1)}\dot{R}=O$. Here, we assume that *A*^(0)^ and *A*^(1)^ are full column-rank matrices and *R* is a general *N*_*R*_ × *N*_*s*_ time-varying matrix. Because $\dot{W}=O$ holds for the fixed point, *W* gives an ICA solution if and only if $W{A}^{(1)}\dot{R}=O$. Thus, *W* needs to satisfy *W*(*A*^(0)^, *A*^(1)^) = (Ω, *O*) to give a multi-context ICA solution. The condition for such an ICA solution to exist was obtained as follows: we considered this as a BSS problem such that

10 $$\begin{array}{c}x=({A}^{(0)},{A}^{(1)})(\begin{array}{c}s\\ Rs\end{array}).\end{array}$$

The singular value decomposition is given by $({A}^{(0)},{A}^{(1)})=US({V}_{0}^{T},{V}_{1}^{T})$, where $U\in {{\mathbb{R}}}^{{N}_{x}\times ({N}_{s}+{N}_{R})}$, ${V}_{0}\in {{\mathbb{R}}}^{{N}_{s}\times ({N}_{s}+{N}_{R})}$, and ${V}_{1}\in {{\mathbb{R}}}^{{N}_{R}\times ({N}_{s}+{N}_{R})}$ with ${V}_{0}{V}_{1}^{T}=O$ are orthogonal matrices and $S\in {{\mathbb{R}}}^{({N}_{s}+{N}_{R})\times ({N}_{s}+{N}_{R})}$ is a diagonal matrix of singular values. From this, *W* = Ω*V*_0_*S*^−1^*X* should hold to ensure *W*(*A*^(0)^, *A*^(1)^) = (Ω, *O*), where *X* is an orthogonal matrix satisfying *XU* = *I*. Hence, ICA solutions exist when and only when column vectors of (*A*^(0)^, *A*^(1)^) are linearly independent of each other.

Moreover, we analyzed a sufficient condition on the time constant of *R*(*t*) for the stability of the ICA solution. From our previous analysis, the linear stability for fixed points is determined by the following second differential form^15,17^:

11 $$\begin{array}{c}{d}^{2}L={(\sum _{i=1}^{{N}_{u}}d{K}_{ii})}^{2}+\sum _{i=1}^{{N}_{u}}\,(1+{{\rm{\Phi }}}_{ii})\,d{K}_{ii}^{2}+\frac{1}{2}\sum _{i=1}^{{N}_{u}}\,\sum _{i\ne j}\,({{\rm{\Phi }}}_{ij}d{K}_{ij}^{2}+2d{K}_{ij}d{K}_{ji}+{{\rm{\Phi }}}_{ji}d{K}_{ji}^{2}),\end{array}$$

where ${{\rm{\Phi }}}_{ii}\equiv {\rm{cov}}[-\mathrm{log}\,{p}_{0}({s}_{i}),g^{\prime} ({s}_{i}){s}_{i}^{2}]$ and ${{\rm{\Phi }}}_{ij}\equiv {\rm{cov}}[-\mathrm{log}\,{p}_{0}({s}_{i}),g^{\prime} ({s}_{i})]{s}_{j}^{2}+{\rm{cov}}[-\mathrm{log}\,{p}_{0}({s}_{j}),{s}_{j}^{2}]g^{\prime} ({s}_{i})$ for *i* ≠ *j* (note that cov[,] is the covariance). The magnitude of *dW* is assumed to be negligible due to a small learning rate. The solution is linearly stable when and only when Φ_*ii*_ > −1 and Φ_*ij*_Φ_*ji*_ > 1. When change in *R*(*t*) is sufficiently slower than that of *s*(*t*) on average, i.e., when *dK* = *dW*(*A*^(0)^ + *A*^(1)^*R*) + *WA*^(1)^ *dR* is sufficiently small, the above linear stability condition determines the stability of the fixed point. However, when *R*(*t*) changes faster than or as fa*st* as *s*(*t*), *dK* is no longer a small fluctuation, because of large *dR*, and therefore *K* may leave from the neighborhood of the fixed point to the region where the second order approximation is no longer accurate. Therefore, as long as the time constan*t* of *R*(*t*) is chosen to ensure the averaged fluctuation is small and thus *K* is within the neighborhood of the fixed point, the EGHR with a time-varying mixing matrix has the same linear stability condition as the conventional EGHR without context switching.

### Analysis of conventional ICA algorithms

Here we show that, unlike the multi-context EGHR, conventional ICA algorithms cannot be used for the dimensionality reduction purpose. Some of the ICA algorithms in consideration are written as $\dot{W}\propto F(u(t),x(t))W$ or, equivalently,

12 $$\begin{array}{c}W(t+1)=[I+\eta F(u(t),x(t))]W(t)\end{array}$$

in each discrete time step *t* (*t* = 1, 2, …, *T*) with learning rate *η*. The functional *F* specifies an individual learning rule, namely, the natural gradient algorithm takes *F*(*u*, *x*) = *I* −〈*g*(*u*)*u*^*T*〉^^12^ and the non-holonomic algorithm takes $F(u,x)=\langle {\rm{d}}{\rm{i}}{\rm{a}}{\rm{g}}[g(u)\odot u]-g(u){u}^{T}\rangle$^39^, where $\odot$ expresses the element-wise product of two vectors and diag[⋅] indicates a diagonal matrix comprising a vector. This class of ICA algorithms cannot perform dimensionality reduction. Following Eq. (12), the synaptic strength matrix after training (i.e., at time *T*) is expressed as

13 $$\begin{array}{c}W=[\prod _{t=1}^{T}\,(I+\eta F(u(t),x(t)))]{W}_{0},\end{array}$$

where *W*_0_ is the initial synaptic matrix. In dimensionality reduction, we are interested in horizontally long *N*_*u*_ × *N*_*x*_ matrices *W* and *W*_0_, which compress *N*_*x*_-dimensional signal *x* to *N*_*u*_-dimensional output *u* with *N*_*u*_ < *N*_*x*_. However, $\prod _{t=1}^{T}(I+\eta F(u(t),x(t)))$ changes the strength only within the *N*_*u*_ × *N*_*u*_ degree of freedom, so that this is equivalent to the ICA of *N*_*u*_-dimensional signals *W*_0_*x* that is already compressed by the non-optimal matrix *W*_0_. Hence, this class of ICA algorithm can be used for separating already (sub-optimally) compressed signals *W*_0_*x* but not for reducing signal dimensions. The infomax-based ICA algorithm^10,11^ has the same fixed point and linear stability conditions as the natural gradient algorithm; thus, again, it does not perform dimensionality reduction. Next, the ICA mixture model was proposed, which is a combination of ICA and a mixture model, to perform multi-context ICA by assigning one of the multiple ICA models to each context^40^. In this model, the pseudo inverse of the synaptic matrix *W*^*k*^ for the ^*k*^-th model is updated instead of *W*^*k*^ by d(*W*^*k*^)^+^/d*t* ∝ *z*^*k*^(*t*)(*W*^*k*^) + (*I* − *g*(*u*(*t*))*u*(*t*)^*T*^), where *z*_*k*_(*t*) ∈ [0, 1] is the probability of the *k*-th model being selected. Similar to Eq. (13), the pseudo inverse of the synaptic strength matrix after training is expressed as

14 $$\begin{array}{c}{({W}^{k})}^{+}={({W}_{0}^{k})}^{+}[\prod _{t=1}^{T}\,(I+\eta {z}_{k}(t)(I-g(u(t))u{(t)}^{T}))],\end{array}$$

which indicates again that the compression is determined by ${W}_{0}^{k}x$. Therefore, the ICA mixture model does not perform dimensionality reduction, either. Hence, the use of multi-context ICA for dimensionality reduction is our novel contribution to the literature, which is beyond the original proposal of the EGHR or conventional multi-context ICA algorithms.

### Simulation protocols

*For figure 5:* Two birdsongs were downloaded from Xeno-canto (https://www.xeno-canto.org/132149, https://www.xeno-canto.org/133054). Two hidden sources were created by trimming the first 60 s of these songs (with 4410-Hz time resolution) and normalizing them, to ensure each source sequence had zero mean and unit variance. During the training, the song sequences were repeated. To add stochasticity, a hidden source was defined by the sum of a song and a white-noise sequence generated by a Laplace distribution. The mixing matrix was defined by 6 × 2 random matrices, *A*^(0)^, *A*^(1)^, and a rotation matrix, $R(t)\equiv (\cos \,\omega t,-\,\sin \,\omega t;\,\sin \,\omega t,\,\cos \,\omega t)$. The angular frequency *ω* was randomly set as −0.1*π*, 0, or 0.1*π* [rad/s], by following Markov process with a transition probability of 1/8820. The training time and learning rate were defined by *T* = 4410 × 6000 [step] and *η* = 10^−7^.

*For figure 6:* Ten birdsongs were downloaded from Xeno-canto (the URLs are https://www.xeno-canto.org/****** where ****** was replaced with the following numbers: 27060, 64735, 67307, 110303, 121326, 121691, 126481, 132149, 133054, 133862). Ten hidden sources were created in the same manner as described above. The mixing matrix was defined by 100 × 10 random matrices *A*^(0)^, *A*^(1)^, *A*^(2)^, *A*^(3)^, *A*^(4)^, where ${\hat{A}}^{(1)},\ldots ,{\hat{A}}^{(4)}$ were randomly generated and *A*^(0)^, …, *A*^(4)^ were defined by ${A}^{(0)}=({\hat{A}}^{(1)}+{\hat{A}}^{(2)}+{\hat{A}}^{(3)}+{\hat{A}}^{(4)})/4$ and ${A}^{(k)}={\hat{A}}^{(k)}-{A}^{(0)}$, for *k* = 1, …, 4. This treatment was served to ensure that *A*^(1)^, …, *A*^(4)^ do not involve common features across contexts. The training comprised 120 sessions, with each session continued for *T* = 4410 × 600 [step]. The context vector *v* randomly chose one of the following ten vectors, *v* = (1,0,0,0), (½,½,0,0), (0,1,0,0), (0,½,½,0), (0,0,1,0), (0,0,½,½), (0,0,0,1), (½,0,0,½), (½,0,½,0), (0,½,0,½), at the beginning of each session and maintained the value during the session. The learning rate was defined by *η* = 2 × 10^−7^. For the test, 20 randomly generated vectors were used, and their elements were randomly sampled from [0,1] and then normalized to satisfy *v*_1_ + *v*_2_ + *v*_3_ + *v*_4_ = 1.

## Supplementary information

Source code 1
